# Association of serum 25-hydroxy vitamin D with gait speed and handgrip strength in patients on hemodialysis

**DOI:** 10.1186/s12882-022-02973-7

**Published:** 2022-11-01

**Authors:** Chen Fu, Fengqin Wu, Fang Chen, Enhong Han, Yuehua Gao, Yongxing Xu

**Affiliations:** 1 Department of Nephrology, Beijing Jishuitan Hospital, 100035 Beijing, China; 2 Department of Nephrology, The Chinese PLA Strategic Support Force Medical Center (The 306th Hospital of Chinese PLA), Beijing, China

**Keywords:** 25-hydroxy vitamin D, Handgrip strength, Gait speed, Hemodialysis

## Abstract

**Background:**

Muscle dysfunction is prevalent in dialysis patients. Gait speed and handgrip strength are simple and reliable methods of assessing muscle function. Numerous observational studies have linked 25-hydroxy vitamin D[25(OH)D] status with gait speed and handgrip strength in populations without kidney diseases. This study aimed to evaluate the potential associations of 25(OH)D status with gait speed and handgrip strength in patients on hemodialysis.

**Methods:**

In this observational cross-sectional study, demographic data, biological data, and dialysis parameters were collected. Gait speed and handgrip strength were measured. Multiple linear regression and logistic regression analysis were used to investigate the relationship of 25(OH)D status with gait speed and handgrip strength after adjusting for potential confounders.

**Results:**

Overall, a total of 118 participants undergoing hemodialysis were included. Seventy-one (60.2%) participants were male. The median 25(OH)D status in participants was 11.58 (interquartile range: 8.51 to 15.41) ng/ml. When controlling for age, gender, dialysis vintage, and other confounders with a p-value < 0.15 in univariate analyses, 25(OH)D was significantly positively associated with gait speed (β = 0.16, 95% CI 0.05 to 0.28, p = 0.006) and handgrip strength (β = 3.83, 95% CI 1.09 to 6.56, p = 0.007).

**Conclusion:**

Our study showed that 25(OH)D status seemed to be associated with gait speed and handgrip strength in patients on hemodialysis. However, these results were not robust. The relationships between 25(OH)D status and gait speed and handgrip should be further explored.

**Supplementary Information:**

The online version contains supplementary material available at 10.1186/s12882-022-02973-7.

## Introduction

Muscle dysfunction is a prevalent condition in dialysis patients [1, 2]. Gait speed and handgrip strength are simple and reliable methods to assess muscle function, which are commonly used [3]. Impaired gait speed and handgrip strength are strongly associated with high mortality in this population [2, 4, 5].

Vitamin D plays a vital role in regulating calcium phosphate homeostasis and mineral bone metabolism; meanwhile, there is emerging evidence that vitamin D is implicated in muscle strength and function [6]. There are several molecular mechanisms of vitamin D’s impact on muscle strength and function [7–9]. Vitamin D modulates muscle cell differentiation, intracellular calcium handling, and genomic activity [6, 9]. The deficiency of vitamin D leads to oxidative stress in skeletal muscle and has an effect on mitochondrial function and the development of skeletal muscle atrophy [8]. Numerous observational studies have linked vitamin D status with gait speed and handgrip in populations without kidney diseases.

Inadequate vitamin D status is common among patients undergoing dialysis [10]. However, the associations of vitamin D status with gait speed and handgrip strength have not been well-established in this population [11, 12]. The relationship between vitamin D status and gait speed was negative according to Kang et al [13]. However, Bucar Pajek et al.[14] found vitamin D status was significantly positively associated with 6-minute walk test results. Some studies focused on the associations between vitamin D status and handgrip strength, and these studies yielded inconsistent results [13–15].

As a result of the conflicting results, this study was conducted to investigate the potential relationship between vitamin D status and gait speed and handgrip strength.

## Materials and methods

### Study participants

This study was designed as a cross-sectional study recruiting patients on hemodialysis from two centers in China (Beijing Jishuitan Hospital and the Chinese PLA Strategic Support Force Medical Center). Ethical approval of this study was obtained from the local Ethics Committee (Ethics Committee of Beijing Jishuitan Hospital, No. 202104-56). All procedures in the study involving human participants were following the ethical standards of the institutional and national research committee and with the 1964 Helsinki declaration and its later amendments or comparable ethical standards. The study was designed following the Strengthening the Reporting of Observational Studies in Epidemiology (STROBE) statement [16].

Adult patients on hemodialysis (aged 18 years and over) were eligible for study participation if they had been treated with hemodialysis for more than three months and could walk independently. Exclusion criteria were as follows: (a) acute cardiovascular and cerebrovascular disease occurred within a month; (b) joint, muscle, neurological, or vascular and other diseases leading to difficulty in completing the tests; (c) native vitamin D supplementation (ergocalciferol, cholecalciferol or calcifediol) within 3 months; (d) pregnant. All participants provided their written informed consent. Patients all received dialysis three times a week. Dialysate A contained sodium chloride, potassium chloride, calcium chloride, magnesium chloride, glacial acetic acid, and an appropriate amount of dialysis water, and dialysate B contained sodium bicarbonate with an appropriate amount of dialysis water. The dialysate flow rate was 500 mL/min, the blood flow rate was 200 to 280 mL/min, and the dialysis duration was 4 h each time.

### Demographic, clinical, and biochemical data

Demographic and clinical data, including age, sex, weight, height, dialysis vintage, causes of end-stage kidney disease, and comorbid conditions were obtained from participant interviews and a review of medical records. We calculated body-mass index (BMI; kg/m²) from dry weight and height.

Blood was collected before and after dialysis. The following biochemical variables were measured: hemoglobin, sodium, potassium, calcium, phosphate, creatinine, urea, intact parathyroid hormone (iPTH), bicarbonate, albumin, and high sensitivity C-reactive protein (hs-CRP) by an autoanalyzer with standard procedures. Albumin-adjusted calcium concentration was calculated using serum albumin and calcium concentrations. The conventional urea-kinetic measure known as single-pool Kt/V (spKt/V) was used to estimate the dialysis dose. In this term, K is dialyzer clearance of urea; t is dialysis time; V is the volume of distribution of urea. The spKt/V was estimated by calculation of Daugirdas single pool equation [17]

spKt/V = -ln(R − 0.008 × t) + (4–3.5R) × UF/W.

where R = post-dialysis/pre-dialysis blood urea nitrogen, t = dialysis hours, UF = pre-post-dialysis weight change, and W = post-dialysis weight.

We used 25-hydroxy vitamin D [25(OH)D] as the marker of vitamin D status. Serum 25(OH)D concentrations were measured in samples taken predialysis by electrochemiluminescence immunoassay (Vitamin D total, Roche Diagnostics, Germany).

### Usual gait speed and handgrip strength measurement

Usual gait speed and handgrip strength were measured prior to a midweek dialysis session. Participants’ usual gait speed was assessed using 6-m walking tests [18]. Each participant was asked to complete two trials with a standing start. Participants were instructed to walk at their usual pace until they reach the finish line. One trained interviewer walking alongside the participants recorded the time from the start line to the stop line using a hand-held stopwatch following standardized procedures. Total course time was converted to meters/second (m/s). Participants could use walking aids if necessary but not the assistance of another person [19].

Handgrip strengths, in kilogram-force, were measured using a Jamar dynamometer in the dominant hand or the non-fistula hand if implanted before a dialysis session according to previous studies [4, 20, 21]. The dynamometer was calibrated before each measurement day. The standard positionings recommended are sitting with 90 degrees of elbow flexion [18]. In order to achieve the best performance, patients were instructed to adjust the dynamometer so it fits comfortably to their hand size by an experienced research staff blinded to all clinical and biochemical data of the patients. The arm should be extended sideways from the body, with the dynamometer facing away. Encouragement was given while doing so. Each trial was repeated three times with intervals of 60 s and the maximum value was used [18].

### Statistical analysis

Characteristics of the study population were reported as mean ± standard deviation, median and interquartile range, or frequencies, as appropriate. Tests for normality were performed using Skewness and Kurtosis. Skewed continuous variables were logarithmically transformed to ensure normality and better fit a linear relationship between the variables. Bivariate analysis was first performed to evaluate the associations of 25(OH)D with gait speed and handgrip strength. Multiple linear regression analysis was used to investigate the relationship of 25(OH)D with gait speed and handgrip strength. Potential confounders with a p-value < 0.15 in univariate analyses were considered, based on previous studies [22, 23]. Model 1 was unadjusted and only included 25(OH)D. Model 2 included 25(OH)D and demographic variables with a p-value < 0.15 in univariate analyses as confounders. Model 3 included variables in Model 2 plus biological data and dialysis variables with a p-value < 0.15 in univariate analyses. Although 25(OH)D status is influenced by climate and season, laboratory and anthropometry data were collected during the same period. Hence, seasonal variations were not entered in adjusted models. Meanwhile, 25(OH)D status was dichotomized at a cutoff point of 10 ng/mL to assess the consistency of our results based on previous studies [24–26]. According to the Asian Working Group for Sarcopenia (AWGS) consensus [18], low handgrip strength is defined as < 28 kg for men and < 18 kg for women, and low gait speed is defined as 6-m walk speed < 1.0 m/s. By using these cut-off values, handgrip strength was dichotomized as low handgrip strength and normal handgrip strength; while, gait speed was dichotomized as low gait speed and normal gait speed. Then logistic regression analysis was performed to analyze the associations of low handgrip strength and gait speed with the 25(OH)D. A 2-sided P value of 0.05 was considered statistically significant. All analyses were done using STATA 15 (StataCorp LP, College Station, TX).

## Results

### Participants characteristics

A total of 206 patients were screened in our hemodialysis center. Finally, 118 patients participated in the study. The basic characteristics of the participants are presented in Table 1. In the present study, 71 (60.2%) participants were male. Causes of end-stage kidney disease were diabetes mellitus (31.4%), glomerulonephritis (28.8%), hypertensive nephrosclerosis (16.1%), and others/undetermined nephropathies (23.7%). The median 25(OH)D status was 11.58 (interquartile range: 8.51 to 15.41) ng/ml in participants. In this study, 40 (33.9%) of participants had serum 25(OH)D status less than 10 ng/ml, 66 (55.9%) had 25(OH)D status between 10 and 20 ng/ml, 9 (7.6%) had 25(OH)D status between 20 and 30 ng/ml, and 3 (2.5%) had a 25(OH)D status above 30 ng/ml. The mean gait speed was 0.95 ± 0.22 m/s (0.93 ± 0.2 for males and 0.98 ± 0.22 for females, Supplementary File S1) and the mean handgrip strength was 27.92 ± 9.12 kg (31.96 ± 8.93 for males and 21.68 ± 4.98 for females, Supplementary File S1).

**Table 1:** Basic characteristics of study participants

Parameters	Total	25(OH)D ≤ 10.0 ng/ml	25(OH)D > 10.0 ng/ml	P value
Men (%)	71 (60.2%)	21 (52.5%)	50 (64.1%)	0.22
Age, year	61.4 ± 12.8	61.5 ± 12.4	61.3 ± 13.0	0.93
BMI, kg/m^2^	23.6 (20.8, 26.2)	24.0 (20.7, 27.1)	23.5 (20.8, 25.9)	0.53
Causes of end-stage kidney disease				0.42
Diabetes mellitus	37 (31.4%)	16 (40.0%)	21 (27.0%)	
Glomerulonephritis	34 (28.81%)	9 (22.5%)	25 (32.0%)	
Hypertensive nephrosclerosis	19 (16.1%)	5 (12.5%)	14 (18.0%)	
Others and Undetermined	28(23.73%)	10 (25.0%)	18 (23.1%)	
Hemoglobin, g/L	112.7 ± 12.2	112.6 ± 12.1	112.8 ± 12.4	0.92
Sodium, mmol/L	140.0 (138.0, 141.9)	139.2 (138.0, 141.4)	140.0 (138.0, 142.0)	0.52
Potassium, mmol/L	5.02 ± 0.76	4.88 ± 0.75	5.10 ± 0.76	0.14
Phosphate, mmol/L	1.53 ± 0.44	1.53 ± 0.43	1.53 ± 0.45	0.97
Bicarbonate, mmol/L	22.71 ± 3.02	23.32 ± 2.60	22.39 ± 3.18	0.11
Albumin-adjusted calcium, mmol/L	2.20 ± 0.20	2.23 ± 0.19	2.19 ± 0.21	0.28
Albumin,g/L	40.91 ± 2.67	40.89 ± 2.51	40.92 ± 2.77	0.94
hs-CRP, mg/L	2.20 (0.99, 6.67)	1.83 (1.04, 5.42)	2.39 (0.96, 6.92)	0.54
Predialysis creatinine, µmol/L	921.6 ± 217.0	854.7 ± 216.5	956.0 ± 210.4	0.02
Predialysis BUN, mmol/L	24.9 (22.8, 27.3)	24.1 (20.8, 26.3)	25.0 (23.1, 27.7)	0.13
iPTH, pmol/L	88.0 (23.2, 200.6)	100.4 (27.6, 171.0)	85.2 (22.2, 207.9)	0.99
25(OH)D, ng/ml	11.58 (8.51, 15.41)	7.49 (5.56, 8.53)	13.73 (11.59, 17.86)	< 0.001
single-pool Kt/V	1.43 (1.24, 1.66)	1.51 (1.29, 1.64)	1.36 (1.21, 1.66)	0.46
Dialysis vintage, months	82 (52,135)	81 (43,136)	83 (52,130)	0.76
Gait speed, m/s	0.95 ± 0.22	0.88 ± 0.21	0.99 ± 0.20	0.009
Handgrip strength, kg	27.92 ± 9.12	24.79 ± 7.10	29.54 ± 9.65	0.007
Low gait speed	69 (58.5%)	28 (70.0%)	41 (52.6%)	0.07
Low handgrip strengh	28 (23.7%)	12 (30.0%)	16 (20.8%)	0.27

Notes: Values are expressed as means ± standard deviation, medians (interquartile range), or numbers (percentages). To convert plasma iPTH in pmol/L to pg/ml multiply by 9.09; to convert 25(OH)D values in ng/ml to nmol/L multiply by 2.5. Abbreviations: BMI, body mass index; BUN, blood urea nitrogen; hs-CRP, high sensitivity C-reactive protein; iPTH, intact parathyroid hormone; 25(OH)D, 25-hydroxyvitamin D

A total of 69 (58.5%) participants had low gait speed and 28 (23.7%) participants had low handgrip strength. We further made comparisons between participants with 25(OH)D ≤ 10.0 ng/ml and 25(OH)D > 10.0 ng/ml. There was a significant difference in predialysis creatinine, gait speed, and handgrip strength between the two groups. In the participants with 25(OH)D ≤ 10.0 ng/ml, 28 (70.0%) had low gait speed and 12 (30.0%) had low handgrip strength. The difference did not reach statistically significant between the two groups.

### Associations between 25(OH)D and gait speed and handgrip strength

**Fig. 1:**
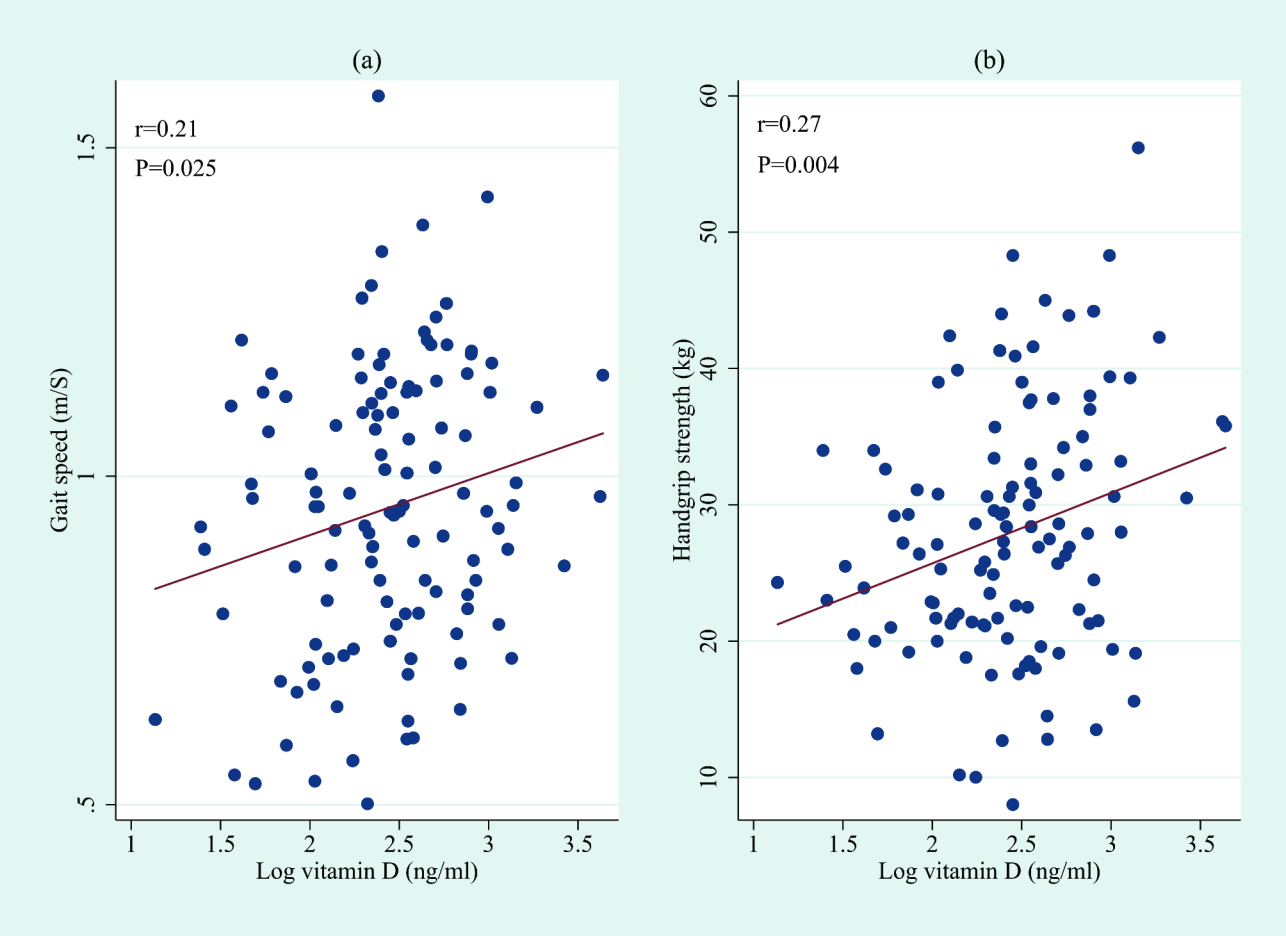
The bivariate analysis showed a significant relationship of vitamin D status with gait speed and handgrip strength. (a) Association between 25(OH)D and gait speed. (b) Association between 25(OH)D and handgrip strength. 25(OH)D had positively skewed distributions and were log-transformed

BMI, dialysis vintage, 25(OH)D, high sensitivity C-reactive protein (hs-CRP), sodium, intact parathyroid hormone (iPTH), and spKt/V with skewed distributions were log-transformed. Bivariate analysis showed that log-transformed 25(OH)D status was significantly correlated to gait speed (r = 0.21, p = 0.025, Fig. 1a) and handgrip strength (r = 0.27, p = 0.004, Fig. 1b).

Multiple linear regression analysis was further performed to investigate the associations of 25(OH)D status with gait speed and handgrip strength (Table 2). In the unadjusted model (Model 1), log-transformed 25(OH)D status was positively correlated to gait speed and handgrip strength. For gait speed, age, and BMI were included in Model 2 according to the results of univariate analyses (Details of univariate analyses are given in Supplementary File S2 and S3); for handgrip, age, gender, and BMI were included in Model 2. After adjusting these parameters, significant associations were still found. In Model 3, laboratory and dialysis parameters with a p-value < 0.15 in univariate analyses were additionally adjusted based on Model 2. For gait speed, albumin, potassium, serum creatinine, and iPTH were additionally adjusted on basis of Model 2. For handgrip strength, albumin, bicarbonate, potassium, corrected calcium, hs-CRP, serum creatinine, and spKt/V were identified as additional confounders. After controlling for all the potential confounders, 25(OH)D remained a significant independent predictor of gait speed (β = 0.16, 95% CI 0.05 to 0.28, p = 0.006) and handgrip strength (β = 3.83, 95% CI 1.09 to 6.56, p = 0.007). When 25(OH)D was dichotomized using a cutoff value of 10 ng/ml in the abovementioned models, the conclusion remained unchanged (Supplementary File S4 and S5). In addition, logistic regression analysis was also performed to analyze the association of low handgrip strength and gait speed with dichotomized 25(OH)D. However, the results of logistic regression analysis were nonsignificant (Supplementary File S6 and S7).

**Table 2:** Associations of 25(OH)D status with gait speed and handgrip strength: multiple linear regression analysis

	Models	β	95% CI	Standard error	P Value
Gait speed	Model 1	0.094^**^	0.012 to 0.177	0.0417	0.025
	Model 2	0.097^**^	0.021 to 0.172	0.038	0.013
	Model 3	0.161^**^	0.048 to 0.275	0.057	0.006
Handgrip strength	Model 1	5.172^**^	1.717 to 8.627	1.744	0.004
	Model 2	4.956^**^	1.851 to 7.291	1.373	0.001
	Model 3	3.825^**^	1.093 to 6.556	1.378	0.007

For gait speed, Model 1: unadjusted model; Model 2: age, BMI, and 25(OH)D; Model 3: Model 2 + albumin, potassium, serum creatinine, and iPTH For handgrip strength, Model 1: unadjusted model; Model 2: age, gender, BMI, and 25(OH)D; Model 3: Model 2 + albumin, bicarbonate, potassium, albumin-adjusted calcium, hs-CRP, serum creatinine, and spKt/V Abbreviations: BMI, body mass index; hs-CRP, high sensitivity C-reactive protein; iPTH, intact parathyroid hormone; spKt/V, single-pool Kt/V; 25(OH)D, 25-hydroxyvitamin D; * P < 0.05; ** P < 0.01

## Discussion

In this observational cross-sectional study, we investigated the associations of 25(OH)D with gait speed and handgrip strength in patients undergoing hemodialysis. The present study suggested that vitamin D status was an independent predictor of gait speed and handgrip strength when gait speed and handgrip strength as continuous variables.

In the current study, we found that vitamin D status seemed to be positively correlated to the usual gait speed in patients on hemodialysis, which has been confirmed in the general population. In the general population, numerous studies have found a positive association between vitamin D and gait speed [27–29]. One recent meta-analysis included 22 studies and provided robust evidence that circulating vitamin D concentrations were positively associated with usual and fast gait speed in the general population [28]. For patients on hemodialysis, Kand and his colleagues’ study [13] showed that the association between vitamin D and gait speed was not statistically significant. By contrast, another study [14] found that vitamin D status was significantly positively associated with 6-minute walk test results, which was consistent with ours.

Some cross-sectional studies examined the association between handgrip strength and vitamin D status in dialysis patients [13–15], and these studies yielded different results. Our result was similar to the study by Kang et al. who found that serum vitamin D is associated with handgrip in multivariate analysis in patients on hemodialysis [13]. By contrast, Bucar and his colleagues [14] showed that no association existed between vitamin D status and handgrip. In another study, Bataille and his colleagues [15] reported a positive association between plasma vitamin D level and handgrip strength. However, Bataille and his colleagues fail to find a significant association when 25(OH)D was analyzed as a continuous variable. In that study, there was a significant association only when 25(OH)D was analyzed as a dichotomous (＜or ≥ 30 ng/mL) variable. The difference might be explained by the different vitamin D statuses of participants. In the study conducted by Bataille et al., the median value was 30.6 (22.5–38.8) ng/ml. In our study, 25(OH)D status was much lower with a median of 11.58 (8.51–15.41) ng/ml and only 3 (2.5%) patients had 25(OH)D status of more than 30 ng/ml. Association might only be true when 25(OH)D was less than 30 ng/ml [15].

Our study found that 25(OH)D was independently related to lower handgrip and gait speed when 25(OH)D was dichotomized into two groups using the cutoff value of 10 ng/ml. For categorical analysis, different cutoff values were used to explore the associations of low 25(OH)D status with health outcomes in chronic kidney disease patients [24–26, 30]. Some studies found that 25(OH)D < 10ng/ml was related to poor outcomes in dialysis patients [24, 25, 31]. So, we adopted the threshold of 10 ng/ml, referring to the previous study [24, 25]. Previous meta-analyses demonstrated a beneficial effect of vitamin D supplementation on muscle strength; this effect is more pronounced in older patients and those with lower 25(OH)D status in the general population [32–34]. Further study should explore the effect of vitamin D supplementation can improve muscle strength, especially in the subgroup with 25(OH)D < 10 ng/ml.

Several mechanisms may explain the link between vitamin D and muscle weakness and poor physical performance. First, vitamin D deficiency may directly affect muscle morphology. Vitamin D deficiency resulted in significant type II muscle fiber atrophy [35]. Moreover, vitamin D deficiency was associated with enlarged interfibrillar spaces and infiltration of fat, fibrosis, and glycogen granules [35]. Vitamin D may also interact with the vitamin D receptor in muscle cells by nongenomic effects. The nongenomic mechanism suggested that the active form of vitamin D acted upon calcium channels on the cell membrane, which in turn led to a rapid influx of calcium into the cell, improving muscle function and contractility [6].

Besides, vitamin D, as a neurosteroid hormone, plays an important role in nervous system function, which may affect gait speed in another way [36]. Vitamin D status was significantly associated with cognitive impairment in both the general population [37] and dialysis patients [38]. Low vitamin D status was associated with worse executive functioning, attention, and nerve conduction velocity, which were involved in gait control [39, 40].

Although some studies have demonstrated the association of vitamin D status with gait speed and handgrip strength, the exact effects of vitamin D supplementation remain uncertain. In a randomized controlled trial [41], participants undergoing hemodialysis were allocated to receive 50,000 IU cholecalciferol or placebo once weekly for eight weeks and then monthly for four months. Over the 6-month follow-up period, no significant differences were found in handgrip strength between the groups. Another self-controlled study [42] included patients on peritoneal dialysis who were prescribed cholecalciferol, 50,000 IU orally once per week for 4–8 weeks. The investigators found that vitamin D supplementation significantly improved handgrip strength from 26.0 to 27.7 kg and gait velocity test from 27.7 ± 3.2 to 25.8 ± 2.9 s. However, the study samples were both relatively small and only 60 and 47 dialysis patients were included, respectively. Furthermore, only 21 patients in the cholecalciferol group (70%) and 24 patients in the placebo group (80%) returned for scheduled 6-month follow-up evaluations in the randomized controlled trial [41]. A high withdrawal rate can lead to bias [43]. The effects of vitamin D on gait speed and handgrip strength still require further exploration.

There were also some limitations in our study. First, the conclusion of this study was not robust. Although the linear regression analysis showed a positive association between 25(OH)D and gait speed and handgrip strength, the logistic analysis yielded nonsignificant results. The nonsignificant results may partially be explained by low power due to the small sample size. Meanwhile, when dichotomizing a continuous outcome to a binary outcome, information is lost and power is reduced [44–46]. Second, when 25(OH)D status was above 30 ng/ml, the association was unknown due to the lower baseline 25(OH)D status in our study. Third, this was an observational study, and patients with native vitamin D supplementation (e.g., ergocalciferol or cholecalciferol) were excluded; consequently, the findings might not indicate a causal relationship. It is still unknown if 25(OH)D is merely a marker or a direct cause of impaired gait speed and handgrip strength. Fourth, a chance finding could not confidently be excluded due to the small sample size. A large-scale randomized controlled study is needed to evaluate the effects of vitamin D administration. Fifth, based on inclusion and exclusion criteria, some patients with certain characteristics were not included. Meanwhile, the participants in our study had a lower 25(OH)D status. Hence, a sampling bias could not be excluded and the study population might not represent all the population of patients on hemodialysis. Sixth, we only examined handgrip strength on the dominant hand, but the maximal handgrip strength may occur on the non-dominant hand. Moreover, data on dietary factors were not collected in our study, and these factors were not adjusted in our regression model. Due to these limitations, we should be cautious about the generalizability of research conclusions.

In conclusion, our study demonstrated that 25(OH)D status was positively associated with gait speed and handgrip strength when gait speed and handgrip strength as continuous variables in patients on hemodialysis. However, further large-scale studies are needed to determine whether a causal relationship exists between 25(OH)D and gait speed and handgrip strength. The exact effects of vitamin D supplementation in dialysis patients should be further explored.

## Practical application

25(OH)D seemed to be associated with gait speed and muscle strength in patients on hemodialysis. In patients with lower 25(OH)D status, sarcopenia should be assessed. In future research, whether there is a cause-effect relationship between them needs to be investigated. Once a cause-effect relationship is confirmed, vitamin D supplementation might offer skeletal health benefits in hemodialysis.

## Electronic supplementary material

Below is the link to the electronic supplementary material.

Supplementary Material 1

## Data Availability

The datasets used and/or analyzed during the current study are available from the corresponding author upon reasonable request.

## Abbreviations

BMI: Body mass index.
BUN: Blood urea nitrogen.
hs-CRP: High sensitivity C-reactive protein.
iPTH: intact parathyroid hormone.
25(OH)D: 25-hydroxyvitamin D.
