# Supplementary material for: Association of serum 25-hydroxy vitamin D with gait speed and handgrip strength in patients on hemodialysis
Source: BMC Nephrol. 2022 Nov 1;23:350. doi: 10.1186/s12882-022-02973-7 (PMC9628114; doi:10.1186/s12882-022-02973-7)
Supplement: Supplementary file 1 — Supplementary Material 1 [file 12882_2022_2973_MOESM1_ESM.docx]

Supplementary File S1. Gait speed and handgrip strength grouped by gender.

| Variables | Male | Female | P Value |
| --- | --- | --- | --- |
| Gait speed, m/s | 0.93±0.21 | 0.98±0.22 | 0.244 |
| Handgrip strength, kg | 31.96±8.93 | 21.68±4.98 | <0.001 |

Supplementary File S2. Univariate analysis for gait speed.

| Variables | β | 95% CI | Std.Error | P Value |
| --- | --- | --- | --- | --- |
| Female | 0.047 | -0.033 to 0.128 | 0.04 | 0.244 |
| Age, year | -0.007 | -0.01 to -0.004 | 0.001 | <0.001 |
| Log-BMI, kg/m^2^ | -0.192 | -0.425 to 0.04 | 0.117 | 0.104 |
| Hemoglobin, g/L | -0.001 | -0.004 to 0.002 | 0.002 | 0.478 |
| Log-sodium, mmol/L | 1.232 | -0.455 to 2.920 | 0.852 | 0.151 |
| Potassium, mmol/L | 0.041 | -0.011 to 0.092 | 0.026 | 0.122 |
| Phosphate, mmol/L | -0.059 | -0.148 to 0.03 | 0.045 | 0.193 |
| Bicarbonate, mmol/L | -0.001 | -0.015 to 0.012 | 0.007 | 0.834 |
| Albumin-adjusted calcium | -0.057 | -0.251 to 0.138 | 0.098 | 0.567 |
| Albumin,g/L | 0.017 | 0.002 to 0.031 | 0.007 | 0.024 |
| Log-hs-CRP, mg/L | 0.001 | -0.026to 0.028 | 0.014 | 0.959 |
| Predialysis creatinine, mg/dL | 0.029 | 0.014 to 0.044 | 0.008 | <0.001 |
| Predialysis BUN, mmol/L | 0.129 | -0.079 to 0.336 | 0.105 | 0.221 |
| Log-iPTH, pmol/L | -0.029 | -0.056 to -0.003 | 0.013 | 0.031 |
| Log-25-hydroxyvitamin D, ng/ml | 0.094 | 0.012 to 0.177 | 0.042 | 0.025 |
| Log-single-pool Kt/V | -0.025 | -0.210 to 0.157 | 0.092 | 0.784 |
| Log-dialysis vintage, months | -0.033 | -0.081 to 0.016 | 0.025 | 0.185 |

Note: Variables with skewed distribution were logarithmically transformed.

Abbreviations: BMI, body mass index; BUN, blood urea nitrogen; hs-CRP, high sensitivity C-reactive protein; iPTH, intact parathyroid hormone.

Supplementary File S3. Univariate analysis for handgrip strength.

| Variables | β | 95 % CI | Std.Error | P Value |
| --- | --- | --- | --- | --- |
| Female (%) | -10.289 | -13.149 to -7.429 | 1.444 | <0.001 |
| Age, year | -0.211 | -0.337 to -0.085 | 0.064 | 0.001 |
| Log-BMI, kg/m^2^ | 12.231 | 2.49 to 21.973 | 4.918 | 0.014 |
| Hemoglobin, g/L | 0.036 | -0.102 to 0.174 | 0.07 | 0.609 |
| Log-sodium, mmol/L | 30.061 | -41.866 to 101.988 | 36.312 | 0.409 |
| Potassium, mmol/L | 1.653 | -0.536 to 3.842 | 1.105 | 0.137 |
| Phosphate, mmol/L | -1.431 | -5.288 to 2.426 | 1.947 | 0.464 |
| Bicarbonate, mmol/L | -0.496 | -1.045 to 0.054 | 0.277 | 0.077 |
| Albumin-adjusted calcium | -8.711 | -16.823 to -0.599 | 4.095 | 0.036 |
| Albumin,g/L | 0.967 | 0.363 to 1.571 | 0.305 | 0.002 |
| Log-hs-CRP, mg/L | -0.897 | -2.041 to 0.247 | 0.578 | 0.123 |
| Predialysis creatinine, mg/dL | 1.906 | 1.315 to 2.497 | 0.298 | <0.001 |
| Predialysis BUN, mmol/L | 0.223 | -0.135 to 0.581 | 0.181 | 0.220 |
| Log-iPTH, pmol/L | -0.295 | -1.444 to 0.854 | 0.58 | 0.612 |
| Log-25-hydroxyvitamin D, ng/ml | 5.172 | 1.717 to 8.627 | 1.744 | 0.004 |
| Log-single-pool Kt/V | -14.581 | -22.064 to -7.098 | 3.777 | <0.001 |
| Log-dialysis vintage, months | -2.273 | -2.405 to 1.858 | 1.076 | 0.800 |

Note: Variables with skewed distribution were logarithmically transformed.

Abbreviations: BMI, body mass index; BUN, blood urea nitrogen; hs-CRP, high sensitivity C-reactive protein; iPTH, intact parathyroid hormone.

Supplementary File S4 Multiple linear regression analysis depicting relationships of 25(OH)D with gait speed when 25(OH)D levels were dichotomized at a cutoff point of 10 ng/mL.

| 25(OH)D | ≥ 10 ng/mL | < 10 ng/mL |
| --- | --- | --- |
| Model 1 | β=0.108 (0.028 to 0.190); *P*=0.009 | reference |
| Model 2 | β=0.104 (0.030 to 0.178); *P*=0.006 | reference |
| Model 3 | β=0.083 (0.008 to 0.157); *P*=0.031 | reference |

Model 1: unadjusted model.

Model 2: age, BMI, and 25(OH)D

Model 3: Model 2+ albumin, potassium, serum creatinine, and iPTH

Abbreviations: BMI, body mass index; iPTH, intact parathyroid hormone; 25(OH)D, 25-hydroxyvitamin D

Supplementary File S5. Multiple linear regression analysis depicting relationships of 25(OH)D with handgrip strength when 25(OH)D levels were dichotomized at a cutoff point of 10 ng/mL.

| 25(OH)D | ≥ 10 ng/mL | < 10 ng/mL |
| --- | --- | --- |
| Model 1 | β= 4.755 (1.330 to 8.181); *P*=0.007 | reference |
| Model 2 | β= 3.688 (0.960 to 6.416); *P*=0.009 | reference |
| Model 3 | β= 3.154 (0.423 to 5.884); *P*=0.024 | reference |

Model 1: unadjusted model.

Model 2: age, gender, BMI, and 25(OH)D.

Model 3: Model 2+ albumin, bicarbonate, potassium, albumin-adjusted calcium, hs-CRP, serum creatinine, and spKt/V

Abbreviations: BMI, body mass index; hs-CRP, high sensitivity C-reactive protein; spKt/V, single-pool Kt/V; 25(OH)D, 25-hydroxyvitamin D;

Supplementary File S6 Multiple logistic regression analysis depicting relationships of 25(OH)D with gait speed when 25(OH)D levels were dichotomized at a cutoff point of 10 ng/mL.

| 25(OH)D | ≥ 10 ng/mL | < 10 ng/mL |
| --- | --- | --- |
| Model 1 | OR=2.1 (0.9 to 4.7); *P*=0.071 | reference |
| Model 2 | OR=2.3 (1.0 to 5.4); *P*=0.062 | reference |
| Model 3 | OR=2.0 (0.8 to 5.0); *P*=0.150 | reference |

Model 1: unadjusted model.

Model 2: age, BMI, and 25(OH)D

Model 3: Model 2+ albumin, potassium, serum creatinine, and iPTH

Abbreviations: BMI, body mass index; iPTH, intact parathyroid hormone; 25(OH)D, 25-hydroxyvitamin D

Supplementary File S7 Multiple logistic regression analysis depicting relationships of 25(OH)D with gait speed when 25(OH)D levels were dichotomized at a cutoff point of 10 ng/mL.

| 25(OH)D | ≥ 10 ng/mL | < 10 ng/mL |
| --- | --- | --- |
| Model 1 | OR=1.6 (0.7 to 3.9); *P*=0.270 | reference |
| Model 2 | OR=2.1 (0.8 to 5.3); *P*=0.130 | reference |
| Model 3 | OR=2.1 (0.7 to 5.9); *P*=0.167 | reference |

Model 1: unadjusted model.

Model 2: age, gender, BMI, and 25(OH)D.

Model 3: Model 2+ albumin, bicarbonate, potassium, albumin-adjusted calcium, hs-CRP, serum creatinine, and spKt/V

Abbreviations: BMI, body mass index; hs-CRP, high sensitivity C-reactive protein; spKt/V, single-pool Kt/V; 25(OH)D, 25-hydroxyvitamin D;
